# Finger tapping to different styles of music and changes in cortical oscillations

**DOI:** 10.1002/brb3.2324

**Published:** 2021-08-22

**Authors:** Elizabeth L. Stegemöller, Thomas D. Ferguson, Andrew Zaman, Paul Hibbing, Patricia Izbicki, Olave E. Krigolson

**Affiliations:** ^1^ Department of Kinesiology Iowa State University Ames Iowa USA; ^2^ Theoretical and Applied Neuroscience Laboratory University of Victoria Victoria British Columbia Canada; ^3^ School of Exercise Science Physical and Health Education University of Victoria Victoria British Columbia Canada; ^4^ Department of Psychology University of Victoria Victoria British Columbia Canada

**Keywords:** electroencephalography, motor system

## Abstract

Music has been a therapeutic strategy proposed to improve impaired movement performance, but there remains a lack of understanding of how music impacts motor cortical activity. Thus, the purpose of this study is to use a time–frequency analysis (i.e., wavelet) of electroencephalographic (EEG) data to determine differences in motor and auditory cortical activity when moving to music at two different rates. Twenty healthy young adults tapped their index finger while electroencephalography was collected. There were three conditions (tapping in time with a tone and with two contrasting music styles), and each condition was repeated at two different rates (70 and 140 beats per minute). A time–frequency Morlet wavelet analysis was completed for electrodes of interest over the sensorimotor areas (FC3, FC4, FCz, C3, C4, Cz) and the primary auditory areas (T7, T8). Cluster‐based permutation testing was applied to the electrodes of interest for all conditions. Results showed few differences between cortical oscillations when moving to music versus a tone. However, the two music conditions elicited a variety of distinct responses, particularly at the slower movement rate. These results suggest that music style and movement rate should be considered when designing therapeutic applications that include music to target motor performance.

## Graphical abstract

This exploratory study sought to understand how music influenced sensorimotor activity. Results revealed that the style of music and movement rate had the greatest impact on differences in sensorimotor cortical oscillations. Thus, music style and movement rate are components to consider in the development of therapeutic strategies using music to improve movement performance.

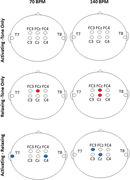

## 1 INTRODUCTION

Music is one of several external cues that have been proposed as therapeutic strategies to improve impaired movement performance in persons with PD (Hackney et al., 2015). For example, dance has been shown to improve functional mobility, gait, and postural instability in persons with PD (Foster et al., 2013; Hackney & Earhart, 2009; 2010; Houston & McGill, 2013; Volpe et al., 2013). Drumming has also been shown to improve gait in persons with PD (Pantelyat et al., 2016). More recently, research has shown that moving with music compared to just a tone reduced movement variability in healthy young adults (Stegemoller et al., 2018). This would suggest that music may provide an added benefit in improving movement performance in persons with PD. Yet, there remains a lack of understanding of how music impacts sensorimotor activity while moving.

The majority of research examining the underlying mechanisms of music have used paradigms in which the participants listen to music. Few studies have examined neural activity while participants move in time with music. Chen and colleagues used functional magnetic resonance imaging (fMRI) to examine auditory–motor interactions, while listening and moving to music have revealed that the premotor cortex plays a role in the processing of rhythm in music (Chen et al., 2006; 2008a; 2008b). However, no comparisons were made to a non‐music condition to determine if their results are uniquely due to moving with music. In addition, fMRI provides spatial information about which sensorimotor regions are involved, but investigations of temporal differences in activity when moving to music is lacking. The use of electroencephalography (EEG) can provide precise temporal information regarding how sensorimotor activity is changing throughout the movement cycle (movement planning to movement execution).

Previous research has shown that sensorimotor activity differs throughout the movement cycle and these differences are modulated by movement rate. During low rate repetitive movements (i.e., discrete movements), cortical activity over the sensorimotor areas has been characterized by a desynchronization of sensorimotor cortical oscillations in the alpha (8–13 Hz) and beta (13–30 Hz) bands right before movement onset and during movement followed by synchronization of oscillations between movements (Erbil & Ungan, 2007; Pfurtscheller & Lopes da Silva, 1999; Stegemöller et al., 2016). In contrast, at higher rate movements (i.e., continuous movement), sensorimotor cortical activity is characterized by a pattern of near continuous desynchronization (Muthukumaraswamy, 2010; Stegemöller et al., 2016; Toma et al., 2002). Two movement rates, one low rate (70 beats per minute [BPM]) and one high rate (140 BPM), were used in this study, as both rates fall within the typical bookends of tempo markings used in music (adagio to allegro), and represent rates at which sensorimotor activity should differ (Stegemöller et al., 2016; Toma et al., 2002). Indeed, our previous work revealed that power spectra when moving with music was increased in the beta band for low movement rates and increased in the alpha band for high movement rates (Stegemoller et al., 2018b). Temporal changes in cortical oscillations when moving to music at high and low rates, to our knowledge, has not been studied. Thus, the purpose of this exploratory study was to use a time–frequency analysis (i.e., wavelet) to determine temporal differences in sensorimotor activity when moving to music compared to a Tone Only at two different rates. We hypothesized that: (1) temporal differences in the beta band will emerge throughout the movement cycle when moving to music as compared to moving to a tone alone, and (2) temporal differences in beta band oscillations will differ at low and high movement rates regardless of cuing condition (music or tone).

**Statement of Significance**

The use of music as a therapeutic is becoming more popular in the treatment of those with Parkinson's disease and other movement disorders. However, there is little understanding of how music impacts sensorimotor activity that contributes to improvements in movement performance in these populations or in healthy populations. This exploratory study is among the first to provide the initial step in understanding how music modulates sensorimotor activity while moving compared to control condition. Results of this study will inform future studies aimed at understanding the use of music to facilitate movement in persons with movement disorders.

## 2 METHODS

### 2.1 Participants

Twenty healthy young adults (11 women, 20 right‐hand dominant, mean ± standard deviation age = 23 ± 3 years) with no history of neurological disorder participated in the study. No intent was made to recruit musicians or non‐musicians for this project, rather a representative population of healthy young adults. However, information about previous music experience was collected. See Table 1 for detailed participant demographics and music experience, the latter being obtained through self‐report. All procedures were approved by the University Institutional Review Board, and all participants signed informed consent prior to data collection. This study was performed in accordance with the ethical standards as laid down in the 1964 Declaration of Helsinki and its later amendments.

**TABLE 1:** Demographic and music experience data for each participant

Subject	Gender	Age	Ethnicity	Instrument	Music experience (years)	Music training (years)
1	F	21	White	Clarinet, Piano	2	2
2	F	21	White	Trumpet, Baritone, voice	5	5
3	F	20	White	Saxophone, Piano, Voice	7	7
4	M	33	Hispanic	Trumpet, Guitar	1	1
5	F	23	Black	None	0	0
6	M	21	White	Piano, Guitar, Voice, Baritone	9	9
7	F	27	White	Piano, Flute, Guitar	9	8
8	M	24	White	Trumpet	2	2
9	F	20	White	Tenor Sax, Clarinet, Flute	8	8
10	F	24	Asian	None	0	0
11	M	22	White	Piano, Guitar, Trumpet, Voice	18	2
12	M	24	Asian	None	0	0
13	M	26	White	Piano, Guitar, Percussion	6	6
14	M	24	White	Trumpet	2	2
15	M	24	White	Tuba, Piano, Guitar, Voice	12	7
16	F	22	White	Guitar, Piano, Banjo, Voice	15	2
17	M	21	White	Trumpet	4	4
18	F	20	White	None	0	0
19	F	28	White	Piano, Clarinet	8	7
20	F	22	White	Flute	12	4

F, female; M, male.

### 2.2 Repetitive movement task

Methods for this study have previously been reported (Stegemoller et al., 2017). In short, participants were instructed to tap their right index finger along with the beat of an acoustic tone and two contrasting forms of music. The dominant forearm and hand were secured in a partial brace in the pronated position. The index finger flexion and extension movements were unconstrained and no tactile feedback was provided. The original pieces of music were composed so no participant had heard it previously. MIDI piano was the only instrumentation and both pieces were composed using part‐writing conventions typical of early 19th‐century Western classical practices. The musical pieces had distinct forms, one featuring an “activating” arrangement while the other featured a “relaxing” arrangement. While participants did not indicate if they thought the pieces of music elicited an activating or relaxing form, these categories are used to distinguish between the two music conditions in this study. See Table 2 for more specifics of the composition of each music condition. A complete description of the music has been previously published (Stegemoller et al., 2017, 2018a). Metronome clicks were inserted in the music conditions to ensure that participants were tapping in time to the same beat as the Tone Only condition. Two paces (70 BPM and 140 BPM) were presented for each condition. This resulted in data being obtained for the following six conditions: (1) Activating at 140 BPM, (2) Activating at 70 BPM, (3) Relaxing at 140 BPM, (4) Relaxing at 70 BPM, (5) Tone Only at 140 BPM, and (6) Tone Only at 70 BPM. Four 10 s trials were completed for each condition, and each condition was randomly presented.

**TABLE 2:** Comparison of music conditions

Activating	Relaxing
C major	G flat major
Ternary form	Through‐composed form
4/4 Meter	3/4 Meter
Harmonic rhythm change every quarter note	Harmonic rhythm change every measure
Buoyant rhythmic patterns and major tonalities	Tonal and metric ambiguities

### 2.3 Data collection

A 2 mm electromagnetic position sensor (Ascension trakStar, Shelburne, Vermont) was placed on the dorsum of the index finger. Electromyography (EMG) sensors were placed on the first dorsal interosseous (FDI) and the extensor digitorum communis (EDC) (Delsys, Natick, Massachusetts). EMG signals were recorded at a sampling rate of 2048 Hz, bandpass filtered in a range from 20–500 Hz, and notch filtered at 60 Hz (The Motion Monitor, Chicago, Illinois). Both the position and EMG data were used to determine movement onset. EEG was also recorded at a sampling rate of 2000 Hz using a 64‐channel unit conforming to the international 10–20 system (Biosemi, Amsterdam, the Netherlands).

### 2.4 Data processing

All EEG data were processed in Matlab using custom code with standard analysis practices (Krigolson, 2018). The code can be found at https://github.com/Neuro‐Tools. Initially, data was inspected and all excessively noisy and/or faulty channels were removed from analysis. The EEG data were then down sampled to 256 Hz and re‐referenced using an average reference. Data were filtered using a dual‐pass Butterworth filter with a passband of 0.1 to 30 Hz and a notch filter at 60 Hz. Following this, a restricted Infomax independent components analysis (ICA) was conducted to identify ocular artifacts (Delorme & Makeig, 2004; Luck, 2014 ). Specifically, for each person we ran the ICA algorithm on continuous (non‐segmented) data. Following this, we then plotted both the ICA component activations across time and the ICA scalp topographic maps. Blinks were identified through manual examination. To identify a blink, we looked for segments where the ICA component activation showed a large deflection in voltage characteristic of a blink. That is, we looked for segments where the change in variation was concentrated in the frontal virtual electrodes of the voltage map where blinks would be expected to be maximal. The ICA approach we used led to the rejection of one component per person on average.

Following ICA, data were then reconstructed using the remaining ICA components. Specifically, following the removal of any ICA components that had the characteristics of a blink, inverse ICA was conducted whereby the matrix of ICA components is multiplied by the mixing matrix. The mixing matrix is simply the inverse of the matrix which was computed by the ICA algorithm to separate the original data into components which were maximally independent (see Makeig & Onton, 2011 for more details). Following the inverse ICA step, all removed channels were interpolated using the method of spherical splines. Any channel that was removed previously due to excessive noise was topographically interpolated through an interpolation algorithm which estimated the removed electrode's activity as per a weighted average of activity of the surrounding electrodes. The method of spherical splines is a form of spline interpolation which works by weighting the electrodes in a manner that best accounts for the dipole fields of the scalp when computing the removed electrode (Ferree, 2006).

EEG data were then segmented using a 1.5 s epoch (−500 to 1000 ms) around movement onset (determined by EMG onset of the FDI) for each condition and were baseline corrected from −500 to −300 ms (the 70 BPM condition) or −300 to −100 (the 140 BPM condition). For each condition, the number of epochs depended on the number of times the participant tapped. As such, for the 140 BPM conditions, this produced an average of 85 epochs per participant per condition (Activating, Relaxing, and Tone Only) while in the 70 BPM conditions this produced an average of 43 epochs per participant per condition (Activating, Relaxing, and Tone Only). Finally, all segments underwent an artifact rejection algorithm that removed segments that had gradients greater than 30 μV/ms and/or a 150 μV absolute within‐segment difference (Luck, 2014). The artifact rejection algorithm led to an average rejection of 9.38% [95% CI: 4.12%, 14.64%] of the total wavelet data for each participant.

A time–frequency wavelet analysis using custom scripts (https://github.com/Neuro‐Tools), adapted from Cohen (2014), was implemented. All time–frequency analyses were conducted on single trials prior to averaging. The time–frequency wavelets were conducted on the pre‐processed, segmented data by multiplying fast Fourier transformed EEG data with complex Morlet wavelets. As per the recommendation of Cohen (2014), we convolved the observed EEG signal with the product of a complex sine wave tapered by a Gaussian window. The convolution window used a 4 ms step size. The number of cycles was varied across each frequency from 3 to 8 cycles. Specifically, the cycle parameter was 3 at 1 Hz, and the cycle parameter increased in a logarithmic manner until reaching 8 cycles at 30 Hz. We chose to vary the number of cycles to appropriately balance the time–frequency precision trade‐off (Cohen, 2014; 2019). In addition, the frequencies of the wavelet were between 1 and 30 Hz, with a step size of 30 linear steps. The window size for the Morlet wavelets was between −500 to 1000 ms, centered around EMG onset for both the Fast (140 BPM) and the Slow (70 BPM) tapping conditions. For the permutation test, we choose a reduced window size of −300 to 800 ms to avoid edge artifacts in the time domain.

The time–frequency wavelets were normalized within each condition through the use of a baseline. The Slow tapping condition (70 BPM) was baseline corrected between −500 and −300 ms pre‐movement onset. In contrast, the Fast tapping condition (140 BPM) was baseline corrected using a window of −300 to −100 ms pre‐movement onset. We choose these separate baselines for the Slow and Fast conditions due to the possible overlap of previous finger taps in the Fast tapping condition if −500 to −300 ms had been used. The baseline procedure was divisive.

### 2.5 Data analysis

A cluster‐based permutation testing was applied to all conditions (Cohen, 2014; see also https://github.com/Neuro‐Tools). In order to compute the permutation test, at each time point and frequency, the average EEG activity across each condition for each participant were computed. We compared the following conditions: (1) Activating and Tone Only, (2) Relaxing and Tone Only, and (3) Activating and Relaxing. This was repeated across both the Slow (70 BPM) condition and the Fast (140 BPM) condition. That is, for each of the three comparisons, we took the participant averages for each condition, and then randomly permutated the condition labels across the averages for each time and frequency. After this, repeated measures *t*‐tests were computed at each point for each permutation. From this random distribution, the most negative and most positive (i.e., most extreme) observations were chosen. Clusters were then defined as continuously significant cells across both time and frequency. This process of extracting the clusters of the random permutations across time and frequency was repeated 1000 times. This led to the creation of a distribution of the most extreme random *t*‐scores (i.e., our null hypothesis) across both time and frequency. Repeated measures *t*‐tests at all time points and frequencies for our own data were completed. That is, using the participant's observed (non‐permuted) average for each of the three comparisons (Activating and Tone Only, Relaxing and Tone Only, Activating and Relaxing), we ran repeated measures *t*‐tests across all time points and frequencies and any clusters that were larger than the 95‐percentile of the null hypothesis from the permutation test were kept. As per above, this was repeated across both the Slow (70 BPM) and Fast (140 BPM) conditions. In order to avoid edge artifacts due to smearing, a permutation window that was between −300 and 800 ms was chosen. After extracting the data, the outputs were *z*‐score corrected for each individual condition and participant by taking each data point (i.e., each time–frequency point) in each condition and subtracting the mean and standard deviation of all conditions and data points. Specifically, this normalization occurred individually for each condition music and pace condition (Relaxing 70 BPM, Relaxing 140 BPM, Activating 70 BPM, Activating 140 BPM, Tone Only 70 BPM, and Tone Only 140 BPM) within each participant.

The cluster‐based permutation test for all six comparisons was completed for the following electrodes of interest: three frontal electrodes (FC3, FCz, FC4), three central electrodes (C3, Cz, C4), and two temporal electrodes (T7, T8) (Muthukumaraswamy, 2010; Stegemöller et al., 2016; Toma et al., 2002). The main comparisons were between the Tone Only condition and the Relaxing condition (Relaxing minus Tone Only) and the Tone Only condition and the active condition (Activating minus Tone Only). Additionally, comparisons were completed between the Activating and Relaxing conditions (Activating minus Relaxing). These comparisons were completed for both the 70 and 140 BPM tapping conditions. However, permutation tests only provide information on whether the difference is significant or not. As such, we used the windows of the permutation test to extract out *p*‐values, means, confidence intervals, and effect size (Cohen's *d*) for all comparisons from the standardized data.

## 3 RESULTS

**FIGURE 1:**
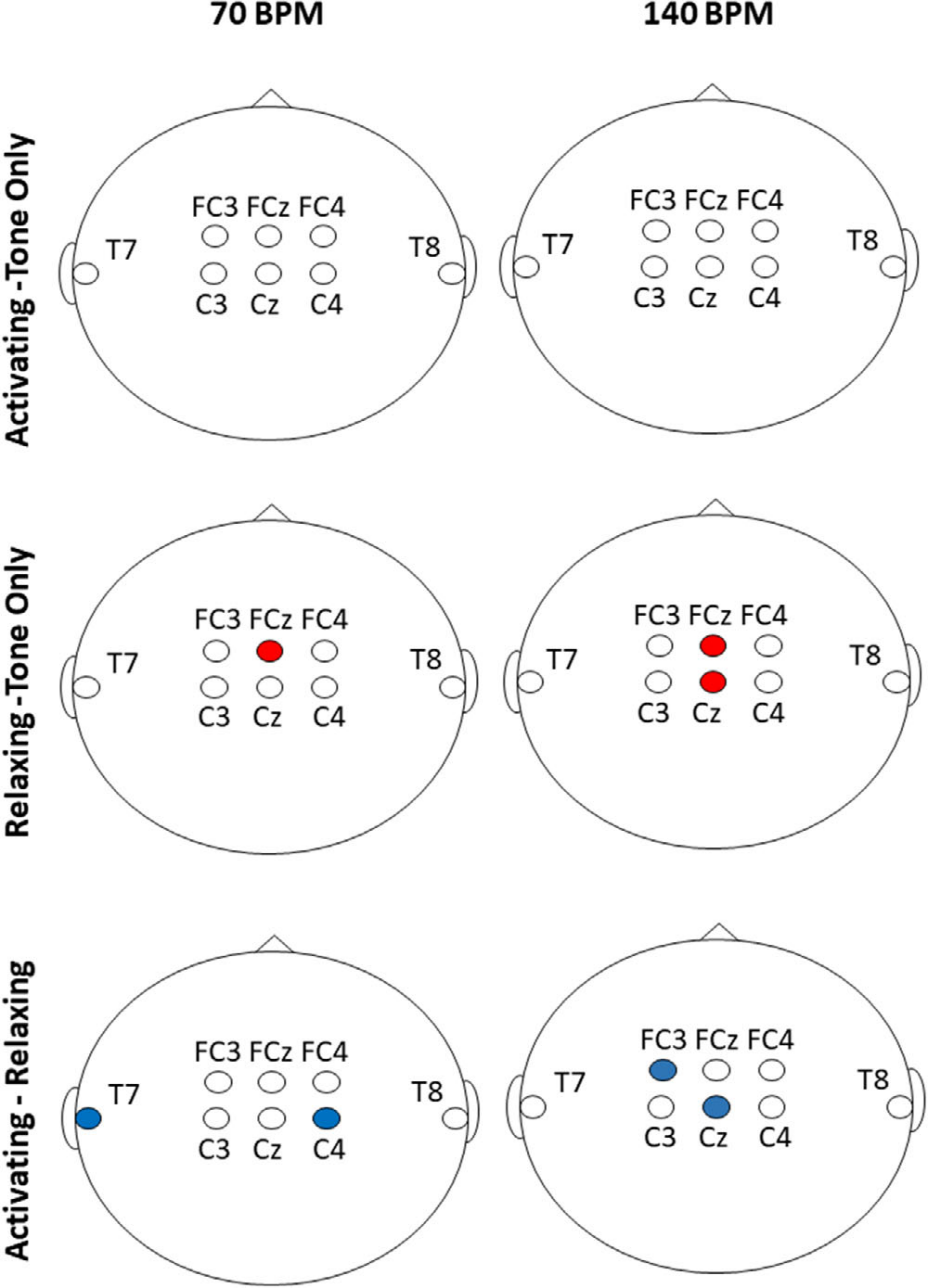
Summary of results for the electrodes of interest for each condition and pacing rate. A red circle indicates higher power and a blue circle indicates lower power for the listed comparisons

Figure 1 summarizes the results of all comparisons. Tables 3 and 4 show individual participant data.

**TABLE 3:** Individual subject averages for each significant permutation test for the Slow (70 BPM) conditions

	Comparison and electrode		
Subject	Relaxing minus Tone Only (FCz)	Activating minus Relaxing (C4)	Activating minus Relaxing (T7)
1	2.87	−3.70	−2.01
2	1.02	−0.09	0.00
3	2.32	0.13	−0.45
4	0.77	0.59	−1.43
5	0.15	−3.28	−5.03
6	2.10	−0.56	−1.52
7	0.22	−0.97	1.17
8	0.51	−2.98	0.18
9	−1.19	−3.54	−5.74
10	0.80	−0.26	0.81
11	2.25	−3.84	−0.42
12	−0.45	−0.40	0.45
13	0.36	1.62	−1.28
14	−1.20	−0.82	−0.31
15	5.83	−4.57	2.59
16	0.97	−1.26	−2.59
17	1.51	−0.16	−3.83
18	0.44	−1.54	−2.08
19	7.58	−1.82	−1.41
20	−0.81	2.89	−1.11

*Note*: Units are in decibels (dB).

**TABLE 4:** Individual subject averages for each significant permutation test for the Fast (140 BPM) conditions

	Comparison and electrode			
Subject	Relaxing minus Tone‐Only (FCz)	Relaxing minus Tone‐Only (Cz)	Activating minus Relaxing (FC3)	Activating minus Relaxing (Cz)
1	0.44	3.42	0.69	1.24
2	0.62	0.55	1.72	−1.32
3	−0.62	0.74	0.46	0.43
4	2.46	1.14	1.46	−2.55
5	−1.61	−1.17	2.15	−3.10
6	0.30	−1.02	−0.13	0.39
7	0.42	3.46	4.16	−2.05
8	0.68	.06	−0.68	−5.29
9	2.31	−0.22	0.35	−1.24
10	1.15	0.66	−0.24	0.07
11	1.64	1.19	−0.09	−3.44
12	2.78	3.23	1.06	−1.95
13	0.23	−0.26	3.85	−2.79
14	0.11	−0.95	−1.50	1.41
15	10.12	5.27	4.68	1.12
16	2.20	2.61	1.21	−1.48
17	0.44	3.28	1.01	0.20
18	−0.54	0.28	0.63	−0.11
19	−0.16	−0.42	0.04	−0.50
20	−0.18	−0.18	−0.57	0.00

*Note*: Units are in decibels (dB).

### 3.1 Activating minus Tone Only

**FIGURE 2:**
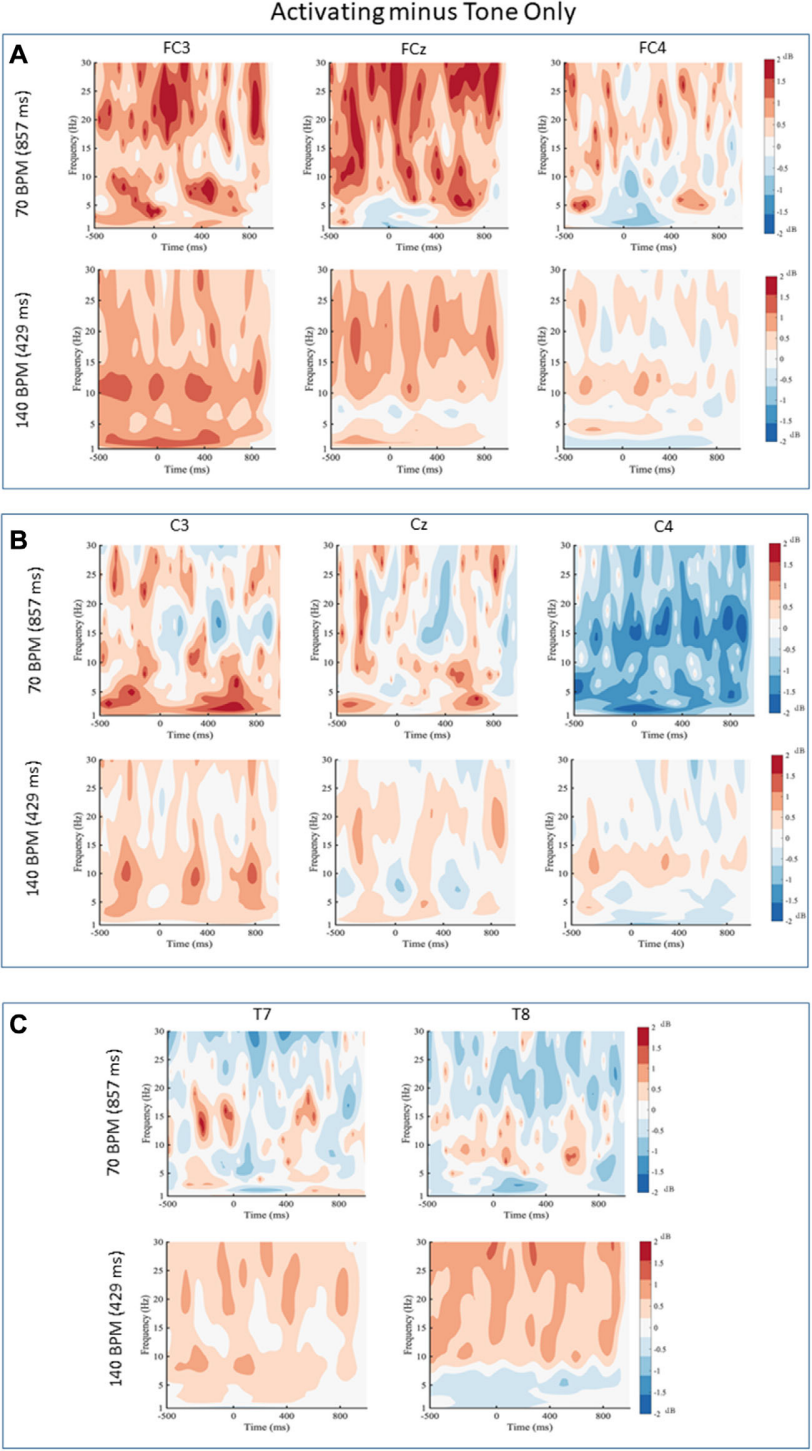
Activating minus Tone Only for (a) frontal electrodes, (b) central electrodes, and (c) temporal electrodes for 70 and 140 beats per minute (BPM). Black contour lines indicate differences that survived the cluster‐based permutation test. Black lines indicate statistical differences in the power spectrum between conditions

The analyses revealed no significant differences between the Tone Only condition and the Activating condition (Figure 2). No differences were revealed for comparisons between the Activating and Tone Only conditions for any of the frontal (FC3, FCz, FC4), central (C3, Cz, C4), or temporal (T7, T8) electrodes.

### 3.2 Relaxing minus Tone Only

**FIGURE 3:**
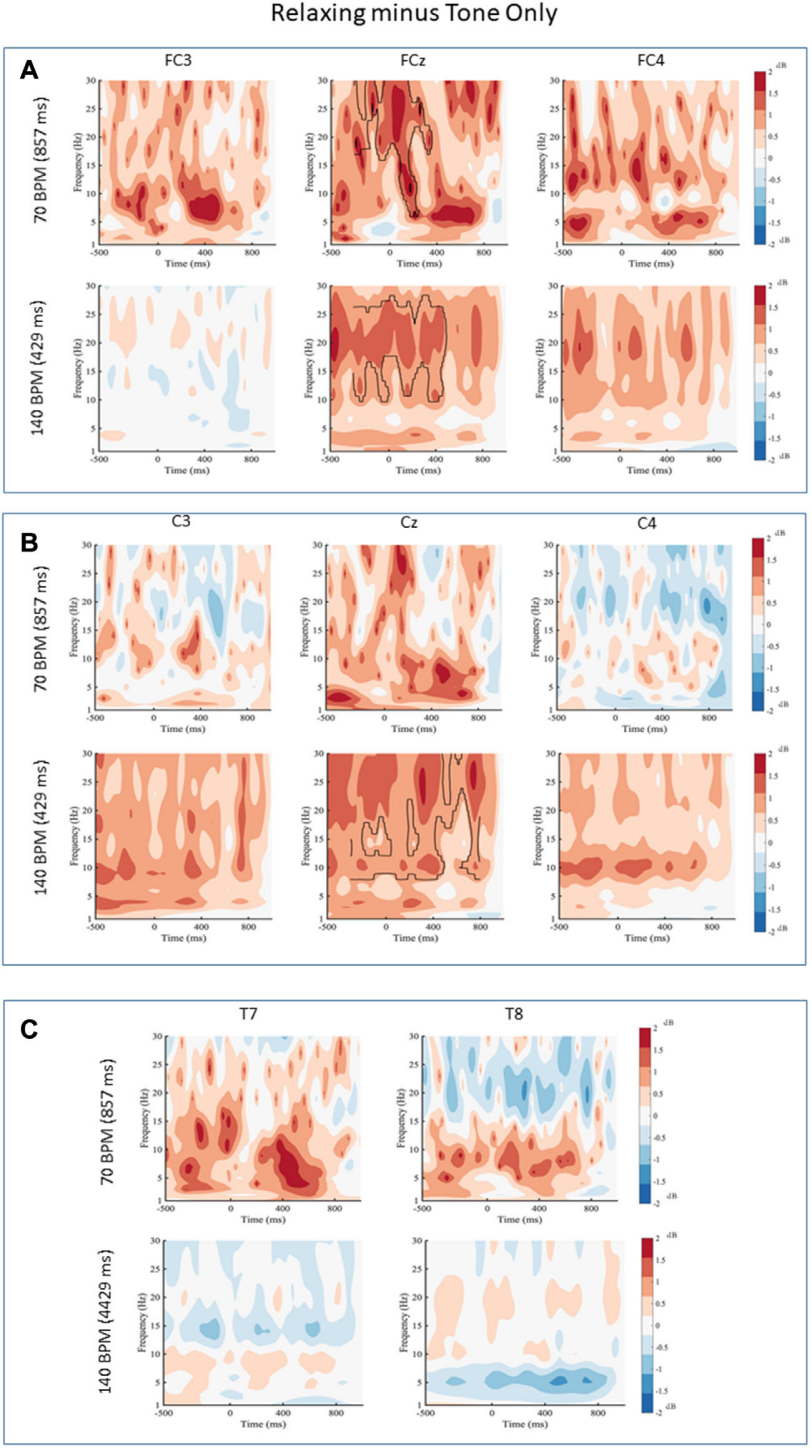
Relaxing minus Tone Only for (a) frontal electrodes, (b) central electrodes, and (c) temporal electrodes for 70 and 140 beats per minute (BPM). Black contour lines indicate differences that survived the cluster‐based permutation test. Black lines indicate statistical differences in the power spectrum between conditions

When comparing the Relaxing and Tone Only conditions, a number of differences were found. More specifically, there was a difference between the Relaxing and Tone Only conditions for 70 BPM over electrode FCz. The Relaxing condition had higher power than the Tone Only condition between 16 to 30 Hz from −300 to 360 ms ( $M_{D}=\;1.30,\;\left[0.28,\;2.32\right],t\;\left(19\right)=\;2.67,\;p<\;.02,\;d\;=\;0.59$). No other differences were revealed for frontal, temporal or the remaining central electrodes within the 70 BPM tapping condition. For the 140 BPM tapping condition, differences were revealed over electrode FCz. The Relaxing condition had higher power than the Tone Only condition between 8 to 30 Hz from −300 to 800 ms ( $M_{D}=\;1.26,\;\left[0.02,\;2.26\right],t\;\left(19\right)=\;2.12,\;p<\;.05,\;\;d\;=\;0.48$). A difference at electrode Cz for the 140 BPM condition was also observed. The Relaxing condition had higher power than the Tone Only condition between 8 and 30 Hz from −300 to 800 ms ($M_{D}\;1.08,\;\left[0.22,\;\;1.93\right],t\;\left(19\right)=\;2.65,\;\;p<\;.05,\;d\;=\;0.59$). No other differences were revealed for central, temporal, or the remaining frontal electrodes at 140 BPM (Figure 3).

**FIGURE 4:**
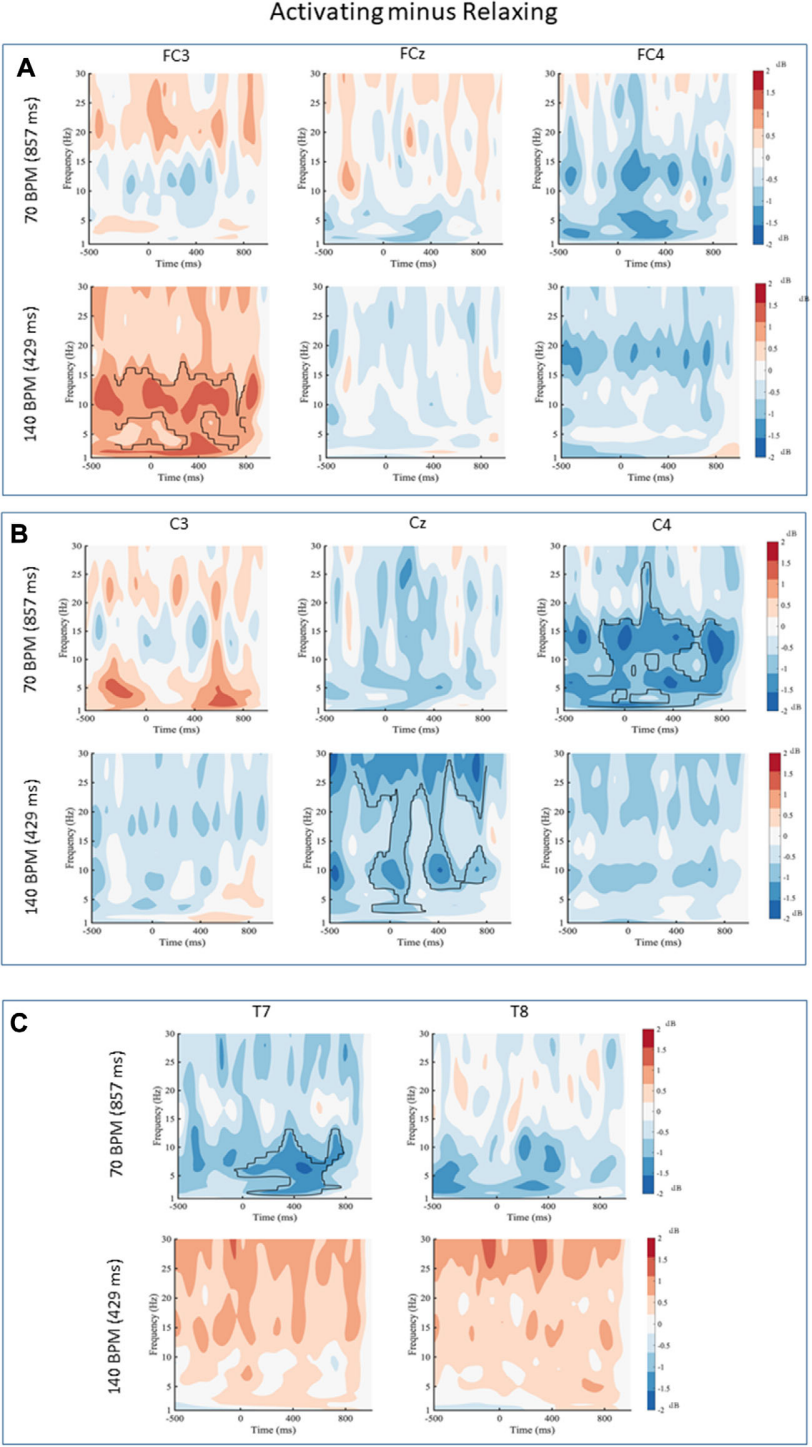
Activating minus Relaxing for (a) frontal electrodes, (b) central electrodes, and (c) temporal electrodes for 70 and 140 beats per minute (BPM). Black contour lines indicate differences that survived the cluster‐based permutation test. Black lines indicate statistical differences in the power spectrum between conditions

### 3.3 Activating minus Relaxing

Finally, the Activating and Relaxing conditions were compared and a number of differences were observed, with the Activating condition generally having lower power than the Relaxing condition. For the 70 BPM condition, differences at electrode C4 were revealed. The Activating condition had lower power than the Relaxing condition between 2 and 27 Hz from −300 to 800 ms at electrode C4 $\left(\;M_{D}=\;-1.23,\;\left[-2.14,-0.32\right],t\;\left(19\right)=\;2.81,\;\;p<\;.02,\;\;d\;=\;-0.58\right)$. In addition, for the 70 BPM music, a difference at electrode T7 was found. The Activating condition had lower power than the Relaxing condition between 2 and 13 Hz from −40 to 788 ms $(\;M_{D}=\;-1.20,\;\left[-2.15,-0.25\right],$ t(19)=2.65,p<.02,d=−0.59). There were no other differences across any of electrodes in the 70 BPM music. For the 140 BPM condition, a number of differences were also revealed. Over electrode FC3, the Activating condition had lower power between 17 to 30 Hz from −300 to 350 ms ($M_{D}=\;1.01,\;\;\left[0.25,\;1.78\right],\;t\;\left(19\right)=\;2.76,\;\;p<\;.05,\;\;d\;=\;0.62).\;$For electrode Cz, the Activating condition had lower power than the Relaxing condition between 3 and 30 Hz from −276 to 800 ms at electrode Cz ( $M_{D}=\;-1.05,\;\;\left[-1.88,-0.21\right],\;t\;\left(19\right)=\;2.62,\;\;p<\;.02,\;\;d\;=\;-0.59).$ There were no other differences for any other electrodes for the 140 BPM condition (Figure 4).

## 4 DISCUSSION

The purpose of this study was to compare the timing of cortical oscillations when moving to music versus moving to a Tone Only at two different rates. Results revealed that timing was mostly unaffected by the experimental condition. For movement to activating music versus a tone, there were no significant differences. For movement to relaxing music versus a tone, differences occurred in the beta band over electrodes FCz (70 and 140 BPM) and the alpha and beta band over electrode Cz (140 BPM). This is in keeping with our previous study that showed a significant increase in beta band power at 70 BPM and a significant increase in alpha band at 140 BPM when comparing both music conditions to the Tone Only condition (Stegemöller et al., 2018). Our previous study examined evoked activity from the motor response by comparing differences across the power spectrum without accounting for changes in power over time. Analyses in this study was intended to capture the single trial activity (including both induced and evoked activity). Thus, the differences revealed may indicate similarities in induced and evoked activity over the sensorimotor cortex when moving with music. However, these results are far from conclusive, and there is still a need for future studies to parse out the effect of music on sensorimotor activity.

Cortical activity differs between low and high rate repetitive movements. During low rate repetitive movements cortical activity is characterized by a desynchronization of oscillations in the alpha and beta followed by synchronization between movements (Erbil & Ungan, 2007; Pfurtscheller & Lopes da Silva, 1999; Stegemöller et al., 2016). At higher rate movements, sensorimotor cortical activity is characterized by near continuous desynchronization (Muthukumaraswamy, 2010; Stegemöller et al., 2016; Toma et al., 2002). Interestingly, in this study most differences in alpha and beta band oscillations recorded over the sensorimotor areas (electrodes C3, C4, Cz, FC3, FC4, FCz) occurred throughout the movement cycle, from roughly 200 ms before to 600 ms after movement onset for both movement rates. Given that the alternating sequence of desynchronization and synchronization of alpha and beta band oscillations is thought to reflect the sensorimotor activity associated with suppression and release of movement (Pfurtsheller et al., 1999), the results of this study may suggest that moving to music may impact both the suppression and release of movement. Yet, there were no differences revealed in cortical oscillations recorded over sensorimotor areas for the activating versus Tone Only condition suggesting that other factors, such as stylistic components of the music were not accounted for. Alternatively, the observed differences in cortical activity could be related to differences due to the participants’ preference in the music used in the present study.

Interestingly, a number of differences were revealed when comparing the two music conditions (i.e., activating versus relaxing music). For those comparisons, significant differences occurred in electrodes FC3 (140 BPM), Cz (140 BPM), C4 (70 BPM),and T7 (70 BPM). Previous research has suggested that music style may impact sensorimotor activity (Janata et al., 2012; Witek et al., 2014; Stegemöller et al., 2018b; Izbicki & Stegemöller, 2020). Faster tempo, moderate syncopation, and repetitive rhythm elicit a greater urge to move while slower tempo, excessive syncopation, and non‐repetitive rhythm elicit little to no urge to move (Janata et al., 2012; Witek et al., 2014). Thus, the two contrasting styles of music used in this study were designed with these details in mind. The intention was that the activating music would elicit a greater urge to move than the relaxing music, which may be reflected in differences in sensorimotor cortical oscillations. Our results revealed that there was a significant decrease in both alpha band and beta band power for multiple electrodes during the Activating condition compared to the Relaxing condition. Given that a decrease or desynchronization in alpha and beta band power may indicate release of movement, the results of this study may indeed support the notion that music style that is designed to increase the urge to move may be reflected in sensorimotor oscillations. However, participants did not indicate if the activating style elicited an urge to move over the relaxing style. Thus, an alternative explanation for differences between the two styles of music may be related to music preference, as we posited above.

The only difference in cortical oscillations recorded over auditory regions emerged when comparing the two music conditions and was in the alpha band. Previous research has suggested that changes in alpha band power over the auditory regions represent a change in listening effort (Marsella et al., 2017; Wisniewski et al., 2017; Wöstmann et al., 2015). A decrease in power may indicate a decrease in listening effort and may suggest that participants in this study displayed more listening effort during the Relaxing condition. Given that the order of the conditions were randomized, the differences may be driven by mechanisms other than fatigue. Given that the activating style was designed with the intent to elicit a greater desire to move than the Relaxing condition, perhaps participants did not need as much listening effort to determine when to synchronize movement. Conversely, the differences in alpha power may be reflective of music preferences in which participants devoted less listening effort to the less preferred style of music. Indeed, participant cohort in this study tended to prefer the relaxing music over the activating music. However, continued research is needed to parse out if the responses revealed in this study are due to differences in the music stimuli or differences in participant factors, such as preference. Nonetheless, results of this study indicate that cortical oscillations over the auditory and sensorimotor areas are influenced by differing styles of music.

## 5 LIMITATIONS

No rating of whether participants perceived the pieces of music as activating or relaxing was recorded. While the intent was for the activating style to elicit more of an “urge” to move than the relaxing style, participant perception of the “urge” to move was not collected. When comparing results to previous studies that carefully describe activating/groovy music, this consideration should be taken into account. The rhythmic and harmonic complexity of the music selections changed throughout the piece. Thus, the auditory content was not the same for each repetitive movement during the music conditions, which in turn may increase the variability in cortical oscillations and may have limited the detection of significant differences. Future studies that address these limitations along with additional coherence analysis will contribute to a better understanding of how music influences motor cortical activity.

## 6 CONCLUSION

This study demonstrated that the style of music may influence alpha and beta band cortical oscillations over both auditory and sensorimotor areas when completing a synchronized finger tap. Moreover, these differences are modulated by movement rate. While these results provide only an initial understanding of how music impacts motor cortical activity, they do suggest the need to consider music style and movement rate when designing therapeutic applications that include music to target motor performance. Future work could build upon our findings by further investigating factors such as the impact of music preference and the impact of listening effort.

## CONFLICT OF INTEREST

The authors declare no competing financial interests.

## AUTHOR CONTRIBUTIONS

Elizabeth L. Stegemöller contributed to study design, data collection, data analysis, writing of first draft, and editing. Thomas D. Ferguson contributed to data analysis, writing of the first draft, and editing. Andrew Zaman, Paul Hibbing, and Patricia Izbicki contributed to data collection, data analysis, and editing. Olave E. Krigolson contributed to data analysis and editing.

### PEER REVIEW

The peer review history for this article is available at https://publons.com/publon/10.1002/brb3.2324

## Supporting information

**Supplemental Figure 1**. Conditional Wavelets for 70 BPM. (a) Relaxing (left) and Tone‐Only (right) at electrode FCz, (b) Activating (left) and Relaxing (right) at electrode C4, (c) Activating (left) and Relaxing (right) at electrode T7.

**Supplemental Figure 2**. Conditional Wavelets for 140 BPM. (a) Relaxing (left) and Move (right) at electrode C3, (b) Relaxing (left) and Move (right) at electrode Cz, (c) Activating (left) and Relaxing (right) at electrode FC3, (d) Activating (left) and Relaxing (right) at electrode Cz.
