# Supplementary figures and images for: Finger tapping to different styles of music and changes in cortical oscillations
Source: Brain Behav. 2021 Aug 22;11(9):e2324. doi: 10.1002/brb3.2324 (PMC8442589; doi:10.1002/brb3.2324)

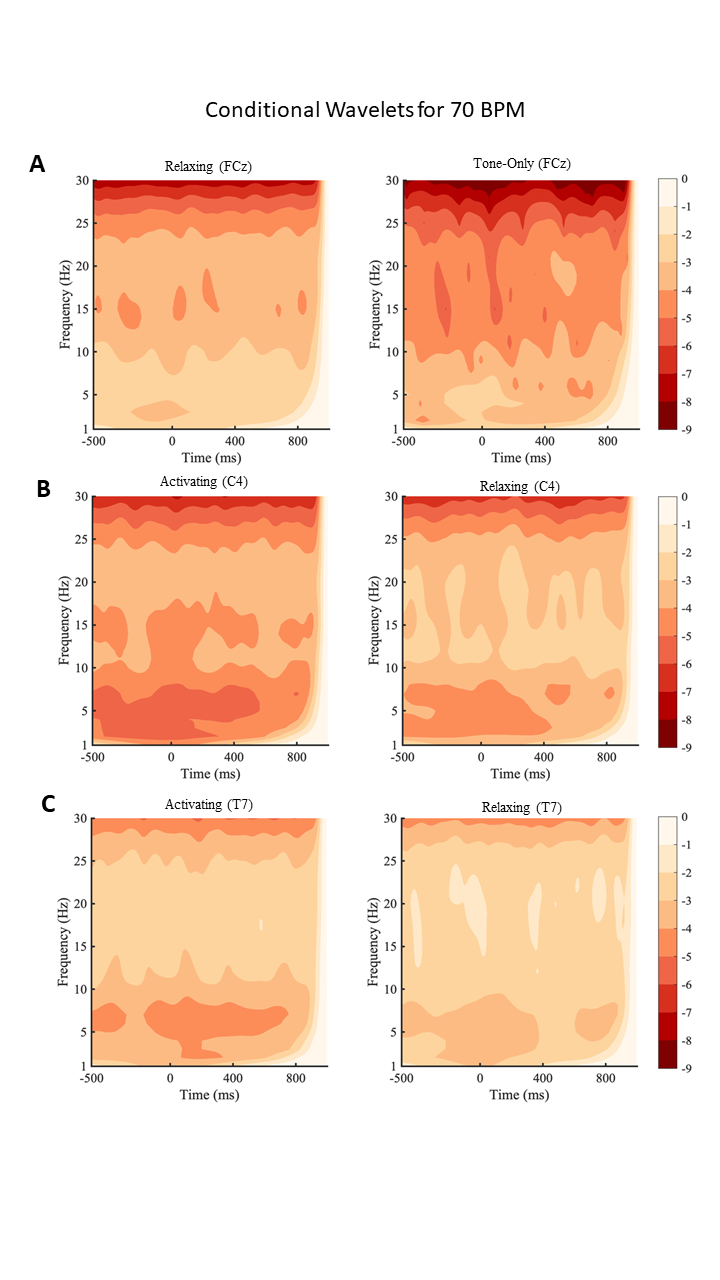

Supplement: Supplementary file 1 — Supplemental Figure 1. Conditional Wavelets for 70 BPM. (a) Relaxing (left) and Tone‐Only (right) at electrode FCz, (b) Activating (left) and Relaxing (right) at electrode C4, (c) Activating (left) and Relaxing (right) at electrode T7. [file BRB3-11-e2324-s002.tif]

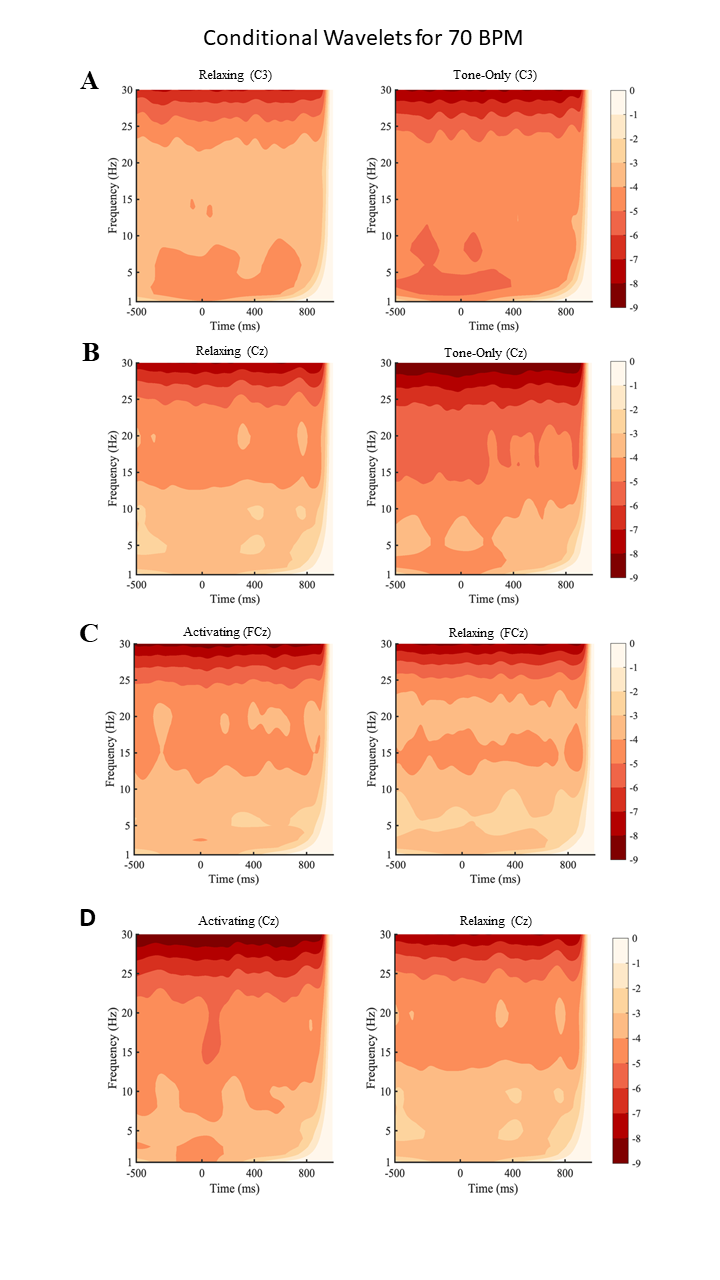

Supplement: Supplementary file 2 — Supplemental Figure 2. Conditional Wavelets for 140 BPM. (a) Relaxing (left) and Move (right) at electrode C3, (b) Relaxing (left) and Move (right) at electrode Cz, (c) Activating (left) and Relaxing (right) at electrode FC3, (d) Activating (left) and Relaxing (right) at electrode Cz. [file BRB3-11-e2324-s001.tif]
